# Supplementary material for: The Construction and Exploration of a Comprehensive MicroRNA Centered Regulatory Network in Foxtail Millet (Setaria italica L.)
Source: Front Plant Sci. 2022 May 6;13:848474. doi: 10.3389/fpls.2022.848474 (PMC9121102; doi:10.3389/fpls.2022.848474)
Supplement: Supplementary file 2 [file Data_Sheet_2.zip › Sit-miRN2306_Seita.5G399600.1_4943_TPlot.pdf]

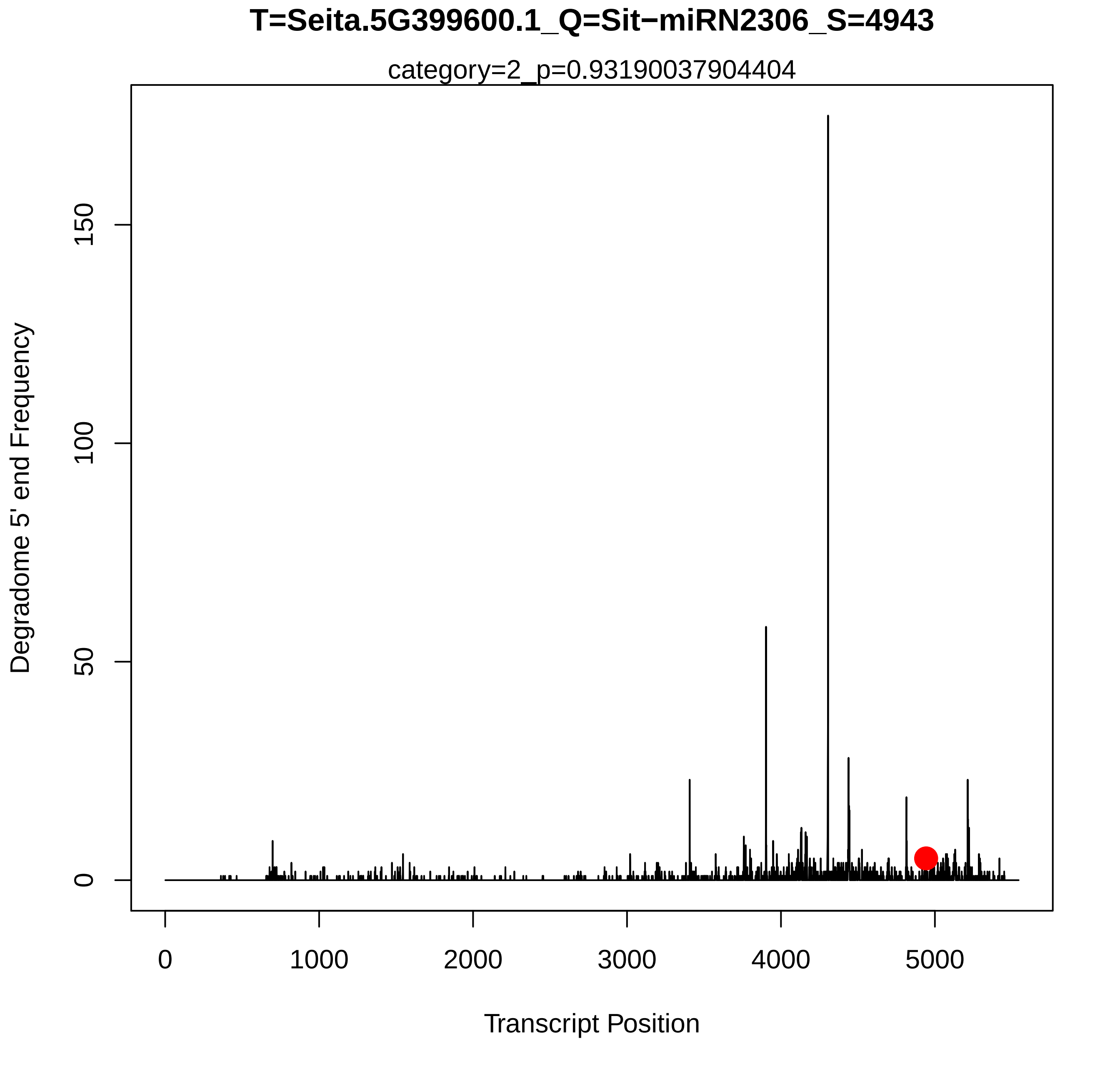
T=Seita.5G399600.1_Q=Sit−miRN2306_S=4943
category=2_p=0.931900379044404
Degradome 5' end Frequency
0
50
100
150
0
1000
2000
3000
4000
5000
Transcript Position
